# λ-Carrageenan P32 Is a Potent Inhibitor of Rabies Virus Infection

**DOI:** 10.1371/journal.pone.0140586

**Published:** 2015-10-14

**Authors:** Zhaochen Luo, Dayong Tian, Ming Zhou, Wenjie Xiao, Yachun Zhang, Mingming Li, Baokun Sui, Wei Wang, Huashi Guan, Huanchun Chen, Zhen F. Fu, Ling Zhao

**Affiliations:** 1 State Key Laboratory of Agricultural Microbiology, College of Veterinary Medicine, Huazhong Agricultural University, Wuhan, Hubei, 430070, China; 2 Glycoscience and Glycoengineering Laboratory, School of Medicine and Pharmacy, Ocean University of China, Qingdao, Shandong, 266100, China; 3 Department of Pathology, University of Georgia, Athens, Georgia, 30602, United States of America; Thomas Jefferson University, UNITED STATES

## Abstract

Rabies, caused by rabies virus (RABV), is an acute, fatal encephalitic disease that affects many warm-blooded mammals. Currently, post-exposure prophylaxis regimens are effective for most rabies cases, but once the clinical signs of the disease appear, current treatment options become ineffective. Carrageenan has been reported as a potent inhibitor of many viruses. In this study, the λ-carrageenan (λ-CG) P32 was investigated for its potential role in inhibiting RABV infection. Our results show that P32 specifically inhibits the replication of several RABV strains but not vesicular stomatitis virus in multiple cell lines and shows low cytotoxicity. P32 mainly abrogated viral replication during the early stage of the post-adsorption period. Further studies demonstrated that P32 could affect not only viral internalization but also viral uncoating by blocking cell fusion mediated by RABV glycoprotein. Moreover, P32 can fully inhibit RABV infection *in vitro* during the post-adsorption period, whereas heparin and heparan sulfate, which possess similar structures to P32, showed significant but not complete inhibition of RABV infectivity. Collectively, our results indicate that λ-CG P32 is a promising agent that can inhibit RABV infection mainly by inhibiting viral internalization and glycoprotein-mediated cell fusion and can be used for the development of novel anti-RABV drugs.

## Introduction

Rabies, caused by rabies virus (RABV), is one of the oldest zoonoses and can infect most warm-blooded animals. RABV is an enveloped, bullet-shaped, negative-stranded RNA virus that belongs to the genus *Lyssavirus* within the *Rhabdoviridae* family. It has been estimated that more than 55,000 people die of rabies and more than 15 million people receive post-exposure prophylaxis (PEP) annually worldwide [1]. Rabies is invariably fatal once the virus reaches the central nervous system (CNS) and neurological symptoms appear [2]. Effective rabies vaccines have been developed; however, prompt vaccination alone may not be sufficient to prevent rabies, and anti-rabies immunoglobulin must also be administered when the exposure is severe. As a result, rabies still poses a public threat in many developing countries in Africa and Asia due to the limited resources and high cost of PEP, especially the anti-RABV immunoglobulin [3].

Few antiviral compounds that are effective against other viruses have been applied to RABV infections [1]. Ribavirin, a broad-spectrum antiviral agent, blocks rabies replication *in vitro* but fails to protect animals or humans from rabies. Interferon-α is effective in RABV-infected monkeys [4] but lacks efficacy in human patients with early rabies encephalitis [5]. In addition, ketamine and ammonium-5-tungsto-2-antimoniate (HPA 23) have been noted for their anti-RABV activity *in vitro* and in rat brain tissue [6, 7], but neither of these compounds has been proven efficacious in human rabies. Thus, no effective therapies for rabies have been identified, and exploring new antiviral drugs is necessary to prevent and reduce the morbidity and mortality of the disease.

Carrageenan is an abundant water-soluble sulfated polysaccharide extracted from red algae that has been widely used in the food industry as an emulsifier, stabilizer or thickener due to its physicochemical stability, safety and affordability [8]. In recent decades, the biological activities of carrageenan have been investigated, including anti-coagulant [9], anti-tumor [10], and immunomodulatory activities [11, 12]. In addition, carrageenan can inhibit a broad range of DNA viruses [13] such as papillomavirus [14, 15] and herpes simplex virus [16–18]. Many RNA viruses, such as influenza A virus, dengue virus, hepatitis A virus and adeno-associated virus type 2, are also sensitive to carrageenan [19–22]. However, whether carrageenan can inhibit RABV has not yet been investigated. In the present study, we investigated the potential role of λ-carrageenan (λ-CG) P32 on the inhibition of RABV infection and found that λ-CG P32 is a promising inhibitor of RABV infection.

## Materials and Methods

### Compounds and reagents

All marine saccharides used in this study were kindly provided by the Glycoscience and Glycoengineering Laboratory at the School of Medicine and Pharmacy of Ocean University of China. Heparin and heparan sulfate (HS) were purchased from Sigma Aldrich (St. Louis, MO, USA) and Iduron (Manchester, UK), respectively. [3-(4, 5-Dimethylthiazol-2-yl)-5-(3-carboxymethoxyphenyl)-2-(4-sulfophenyl)-2H-tetrazolium, inner salt] (MTS) was purchased from Promega Co. (Madison, WI, USA). Dynasore and fluorescein isothiocyanate (FITC)-conjugated anti-RABV N protein antibody were purchased from Sigma Aldrich (St. Louis, MO, USA) and Fujirebio Diagnostics (Malvern, PA, USA), respectively.

### Cells and viruses

Human neuroblastoma (SK-N-SH) cells were purchased from cell bank in the Chinese Academy of Sciences and maintained in minimum essential medium (MEM, Gibco, Grand Island, NY) containing 10% fetal bovine serum (FBS, Gibco, Grand Island, NY). BHK-21 cells and BSR cells (a BHK-21 clone) [23] were maintained in Dulbecco’s Modified Eagle Medium (DMEM, Gibco, Grand Island, NY) supplemented with 10% FBS. Neuroblastoma (NA) cells [24], Vero and HEK-293T cells (purchased from cell bank in the Chinese Academy of Sciences) were cultured in RPM1640 medium (Gibco, Grand Island, NY) containing 10% FBS.

Two recombinant RABVs expressing enhanced green fluorescent protein (eGFP), LBNSE-eGFP and rB2c-eGFP, were constructed based on the recombinant RABVs SAD-L16 (previously designated as LBNSE) [25] and CVS-B2c (rB2c) [26], respectively. A wild-type RABV strain DRV-AH08, isolated from a rabid dog in Anhui province, China, was propagated in the brains of suckling mice [27]. VSV-eGFP (recombinant vesicular stomatitis virus expressing GFP, a gift from Dr. Mingzhou Chen, Wuhan University, China) was propagated in BHK-21 cells.

### MTS method

The cytotoxicity of a λ-CG P32 was analyzed using the MTS method following the manufacturer’s instructions. Briefly, monolayers of different cells (SK-N-SH, HEK-293T, BSR and NA) were prepared in 96-well plates and then incubated with 100 μL of maintenance medium (MM) containing different concentrations (1000, 500, 250, 125, 62.5, 31.25, 15.625 μg/mL) of P32 at 37°C for 48 h. After incubation, 20 μL of MTS solution was added and incubated for 1 h at 37°C. Absorbance at 490 nm was then recorded using an automated plate reader (SpectraMax 190, Molecular Devices, Inc., California, USA). The percentage of cell viability was expressed by comparing λ-CG-treated and mock-treated cells.

### Virus titration

Different RABV strains, including lab attenuated RABV strains (SAD-L16 and rB2c) and a wild-type RABV strain (DRV-AH08), were titrated using a direct fluorescent antibody assay as described previously [28]. Briefly, NA cells in 96-well plates were inoculated with serial 10-fold dilutions of virus and incubated for 48 h at 34°C. After incubation, cells were fixed with 80% ice-cold acetone and then stained with FITC-labeled rabies virus N protein-specific antibodies. Antigen-positive foci were counted using a fluorescence microscope, and viral titers were calculated as focus-forming units/mL (FFU/mL).

For VSV virus titration, viral titers were determined by standard plaque assay using BHK cells. Briefly, the number of plaque-forming units (PFU) was counted in 12-well plates at 24 hpi, and the titer was expressed as PFU/mL.

### Viral inhibition assay

To determine the inhibition efficiency of a λ-CG P32 for viral infection, cells were incubated with MM containing different concentrations of P32 at 37°C for 2 h, after which the medium was discarded and the cells were infected with RABV or VSV at an MOI of 0.01 for 1 h. After incubation, the supernatant was removed, and MM containing the indicated concentrations of P32 was added to the appropriate wells. After incubating the samples at 37°C in a humidified atmosphere of 5% CO_2_ for 48 h (RABV) or 24 h (VSV), the culture medium was harvested for virus titration.

### Viral endocytosis assay

NA cells and BSR cells were infected with SAD-L16 at an MOI of 50 and 0.01, respectively. After incubating the cells for 1 h at 4°C, during which the virus can only adsorb to but not enter into cells [29], the cells were washed with PBS and incubated at 37°C for 1 h in MM with or without P32 or dynasore. The cells were then washed with PBS and treated with proteinase K for 45 min at 4°C to remove adsorbed but not internalized viruses. A cocktail (Roche, Meylan, France) was added to inactivate proteinase K (Sigma-Aldrich, St. Louis, MO, USA), and the cells were washed with PBS containing 0.2% BSA. Total RNA was then extracted from the NA cells using TRIzol (Life Technologies, Inc., Grand Island, NY), and the amount of internalized viral RNA was quantified by qRT-PCR. The virus titration and immunofluorescence assay were carried out in BSR cells to determine the viral titer and RABV N protein levels, respectively.

### Post-internalization inhibition assay

NA cells were infected with SAD-L16 at an MOI of 0.01, and a similar procedure to the viral endocytosis assay was followed with slight modifications. After inactivation of proteinase K, the cells were treated with MM containing P32 (250 μg/mL) or ammonium chloride (20 μM) at 37℃ for 48 h. After incubation, the supernatants were harvested for virus titration.

### Quantitative real-time reverse transcription PCR (qRT-PCR)

The ABI Prism 7500 fast sequence detector system (Applied Biosystems, Inc. Foster City, California, USA) was used to perform qRT-PCR. Total RNA was extracted from RABV-infected SK-N-SH cells using TRIzol (Invitrogen Inc., California, USA). The reverse transcriptase and DNA polymerase used for the reaction were included in the one-step Brilliant II SYBR green qRT-PCR master mix kit (Stratagene, La Jolla, CA). Amplification was performed at 50°C for 2 min and 95°C for 10 min, followed by 40 cycles of 95°C for 15 s and 60°C for 1 min. The primers for RABV viral RNA detection were as follows: forward 5’-GAGGAATTCTTCGGGAAAGG-3’ and reverse 5’-CCTCGTCGTCAGAGTTGACA-3’. The primers for the detection of β-actin (internal control) mRNA were as follows: forward 5’-CCTGTGGCATCCACGAAACTA-3’ and reverse 5’-TGTCAAGAAAGGGTGTAACGCAA-3’.

### Western blotting

Membranes were incubated with polyclonal rabbit antibodies directed against RABV and a mouse anti-β-actin monoclonal antibody, followed by horseradish peroxidase (HRP)-conjugated anti-rabbit and anti-mouse immunoglobulin G. Signals were acquired and analyzed using a Syngene G-Box (Syngene, Frederick, MD).

### Immunofluorescence assay

Cells were fixed with 80% ice-cold acetone and stained with a FITC-conjugated anti-RABV N protein antibody at 37°C for 1 h. After washing three times with PBS, the cells were incubated with 4', 6-diamidino-2-phenylindole (DAPI, Sigma, St. Louis, MO, USA) for 5 min at room temperature and then washed three times. FITC fluorescence was detected using an Olympus IX73P2f fluorescence microscope.

### Low pH-dependent cell fusion assay

A cell fusion assay was performed as previously described [30]. Briefly, BSR cells were infected with SAD-L16 at an MOI of 5 at 37°C for 48 h, which allows for the abundant expression of RABV glycoprotein on the cell surface to trigger membrane fusion under low pH conditions. Cells were treated with MM containing P32 at 37°C for 2 h, after which the cells were washed and incubated with fusion medium [10 mM Na_2_HPO_4_-10 mM NaH_2_PO_4_-150 mMNaCl-10 mM 2-(N-morpholino) ethanesulfonic acid, adjusted to pH 5.6] for 2 min at room temperature. After incubation, the fusion medium was discarded, and MM was added to the wells. After incubation at 37°C for 2 h, the cells were fixed with 80% ice-cold acetone and stained with fuchsine solution [31]. Cell fusion was observed under a light microscope (Zeiss Primo Vert).

### Statistical analysis

All data were analyzed using GraphPad Prism software (GraphPad Software Inc., CA). An unpaired two-tailed t-test was used to determine the statistical significance of the viral titers and the levels of viral RNA in the cell lines.

## Results

### λ-CG can inhibit RABV infection

**Fig 1:**
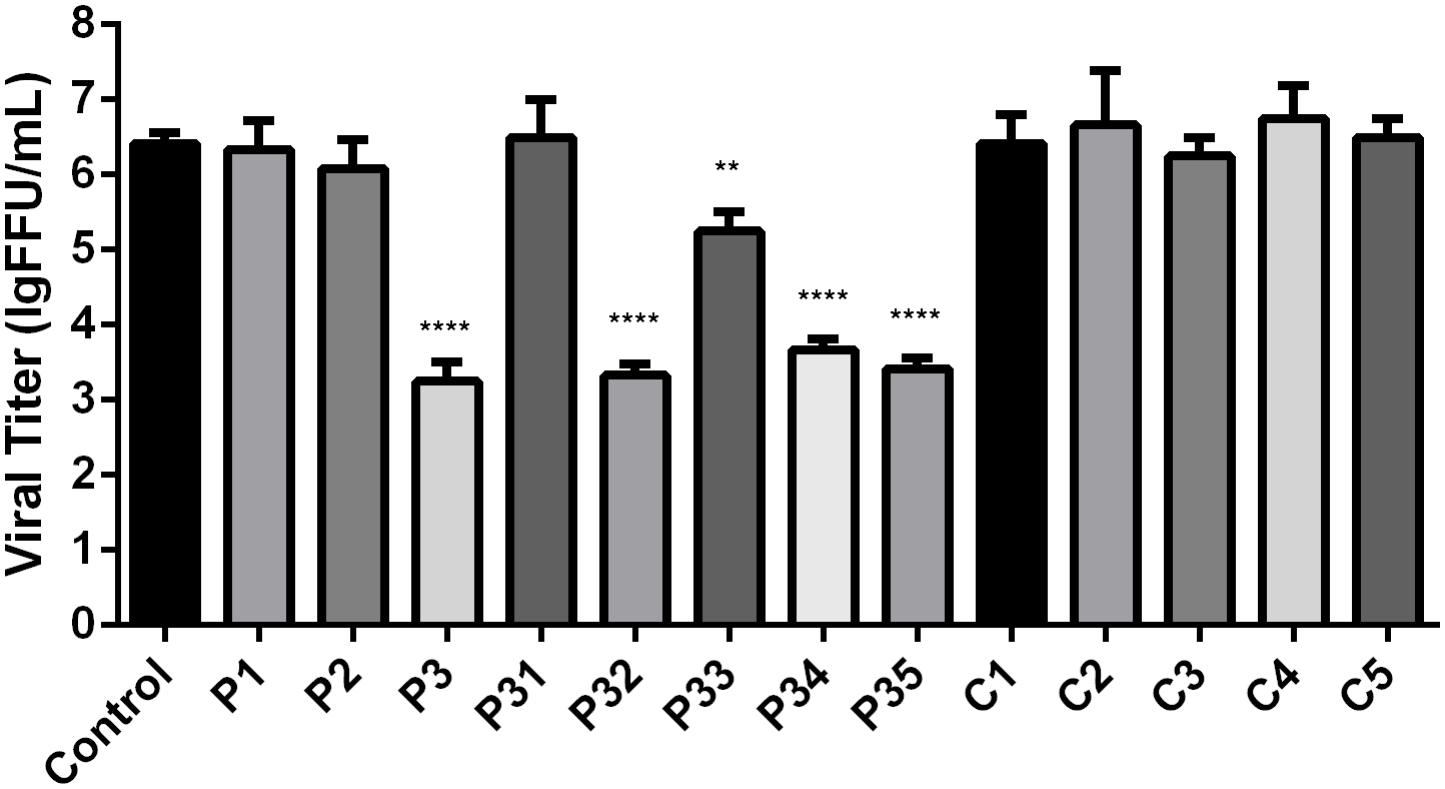
Inhibition effects of thirteen marine saccharides on RABV infection. BSR cells were incubated with different marine saccharides (250 μg/mL) for 2 h, after which they were infected with SAD-L16-eGFP at an MOI of 0.01. After incubation with the virus for 1 h, the cells were washed and maintained in medium containing the same marine saccharide (250 μg/mL) for 48 h. Supernatants were harvested for virus titration. Each value is expressed as the mean ± SEM from three independent experiments. (**, P<0.01; ****, P<0.0005).

To investigate whether marine saccharides could inhibit RABV infection, a drug screen was performed using thirteen different marine saccharides, which could be classified into two categories: chitosans (C1-5) and carrageenans (P1-3 and P31-35). BSR cells were incubated with different compounds (250 μg/mL) for 2 h, after which the cells were infected with RABV strain SAD-L16-eGFP at a multiplicity of infection (MOI) of 0.01. At 48 hpi, viral titers in the supernatant were measured. As shown in Fig 1, five carrageenan saccharides (P3, P32, P33, P34 and P35) could significantly inhibit the infection of SAD-L16-eGFP, with viral titer decreases of approximately 10-fold and 1000-fold for P33 and the other four carrageenan saccharides, respectively. All five carrageenan candidates, including oligosaccharides (P32) and polysaccharides (P3, P33, P34 and P35), belonged to the λ-carrageenan (λ-CG) family. The molecular weights of these λ-CGs are between 4 kDa and 350 kDa, as listed in Table 1. P32, a λ-CG oligosaccharide with the highest inhibitory effect, a low molecular weight (4 kDa), high solubility and high stability, was selected to further investigate the mechanism of λ-CG inhibition of RABV infection.

**Table 1:** Molecular weights of different λ-carrageenans.

Compound Name	P3	P32	P33	P34	P35
Molecular Weight (kDa)	300	4	10	30	350

### P32 shows no obvious cytotoxicity and inhibits RABV replication in a dose-dependent manner in vitro

**Fig 2:**
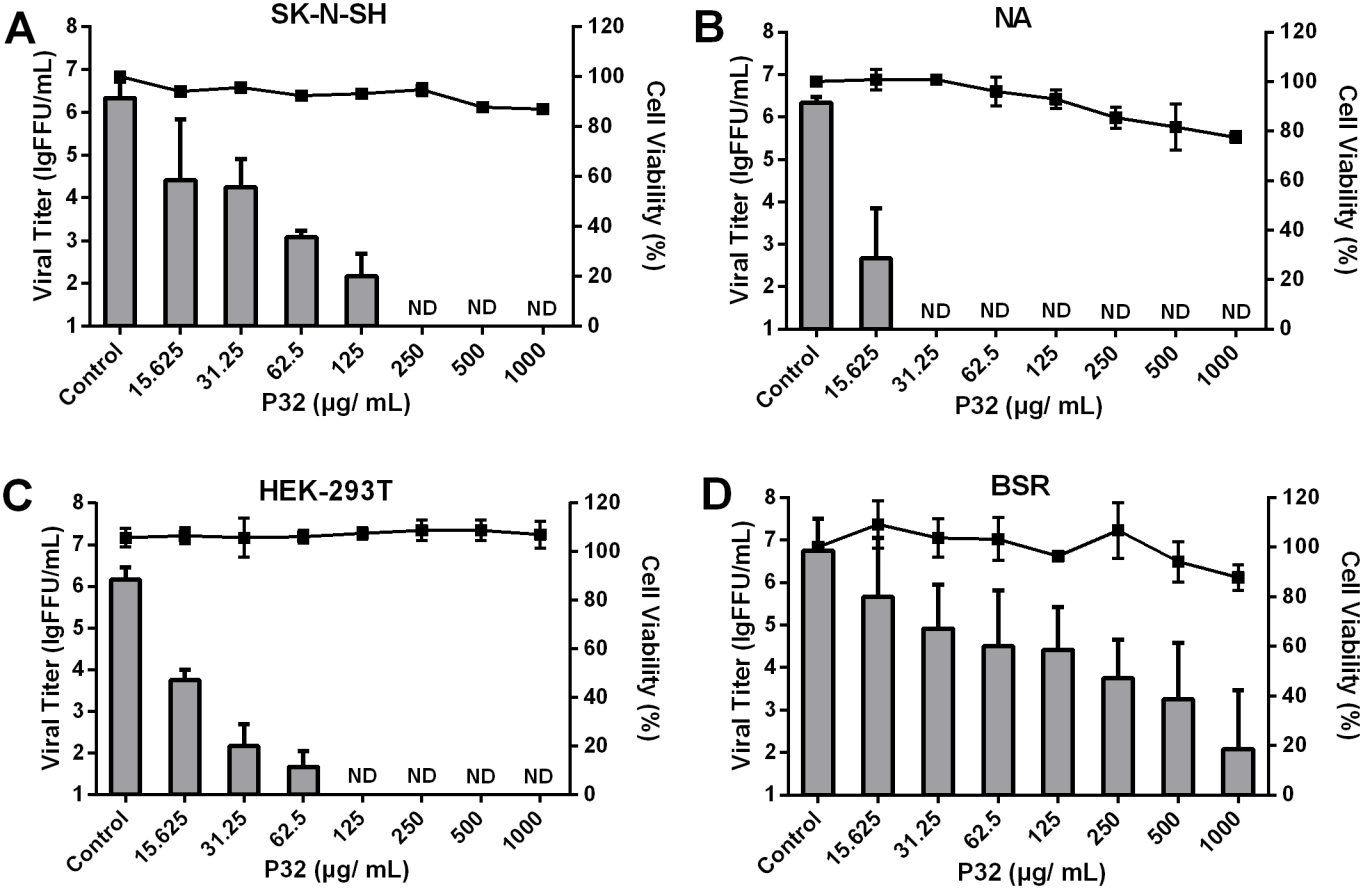
Cytotoxicity and RABV inhibitory effect of P32 on different cell lines. To assess the cytotoxicity of P32 in different cell lines, we used the MTS method. SK-N-SH (A), NA (B), HEK-293T (C), and BSR cells (D) were incubated with different concentration of P32 for 48 h at 37°C, after which the cells were incubated with MTS solution for 1 h. Absorbance at 490 nm was determined, and the percent cell viability for each P32 treatment group was determined by comparing the treated cells with mock-treated cells. For the inhibition assay, cells were infected with SAD-L16-eGFP at an MOI of 0.01 after a 2-h incubation with P32. Supernatants were harvested for virus titration at 48 hpi. Columns and black solid squares represent the viral titers and the cell viability percentages, respectively. Each value is expressed as the mean ± SEM from three independent experiments. ND means non-detectable.

To assess the cytotoxicity of P32, the MTS method was performed on SK-N-SH, NA, HEK-293T and BSR cells. As shown in the right X-axis of Fig 2, no obvious cytotoxicity of P32 was observed at the indicated concentrations, suggesting that P32 could be used for *in vitro* studies. In addition, the inhibitory effect of P32 on SAD-L16-eGFP replication in different cell lines was assessed using the viral inhibition assay. As shown in the left X-axis of Fig 2, viral titer reductions induced by P32 were observed in all tested cell lines in a dose-dependent manner, although the inhibition level varied in different cell lines. In NA, HEK-293T and SK-N-SH cells, RABV replication was completely inhibited with 31.25, 125, and 250 μg/mL P32, respectively. In contrast, the viral titer was deceased by 1000-fold in the presence of 250 μg/mL P32 in BSR cells, although viral replication could not be inhibited in these cells even with as high as 1000 μg/mL P32. Furthermore, the CC_50_ (concentration required to reduce the viability of a given cell line by 50%) and the IC_50_ (concentration required to reduce the viral titer in a given cell line by 50%) were also determined to assess the inhibitory effect of P32 on RABV replication. As shown in Table 2, the CC_50_ values for all cell lines were higher than 1000 μg/mL, and the IC_50_ values for HEK-293T, SK-N-SH, NA and BSR cells were 15.89, 19.93, 22.10 and 57.70 μg/mL, respectively. Therefore, the selectivity index (SI) values are higher than 62.93, 50.18, 45.25, and 17.33 for HEK-293T, SK-N-SH, NA and BSR cells, respectively. Higher SI values indicate stronger inhibitory effects of a compound on viral replication. Therefore, the strongest and weakest inhibitory effects of P32 on RABV replication occur in HEK-293T and BSR cells, respectively.

**Table 2:** Inhibitory effects of P32 on RABV replication in different cell lines.^a^.

Cell line	CC_50_(μg/mL)^b^	IC_50_ (μg/mL)^c^	SI^d^
SK-N-SH	>1000	19.93	>50.18
NA	>1000	22.10	>45.25
HEK-293T	>1000	15.89	>62.93
BSR	>1000	57.70	>17.33

^a^ The inhibitory effects of P32 on SAD-L16-eGFP (MOI = 0.01) replication in these cells were evaluated by viral titer reduction.

^b^ Cytotoxic concentration 50% (CC_50_): Concentration required to reduce the cell viability of a given cell line by 50%.

^c^ Inhibition concentration 50% (IC50): Concentration required to reduce the viral titer of a given cell line by 50%.

^d^ SI: Selectivity index is defined as the ratio of CC50 to IC50 (SI = CC50/IC50).

### P32 inhibits attenuated and wild-type RABV but not VSV replication

**Fig 3:**
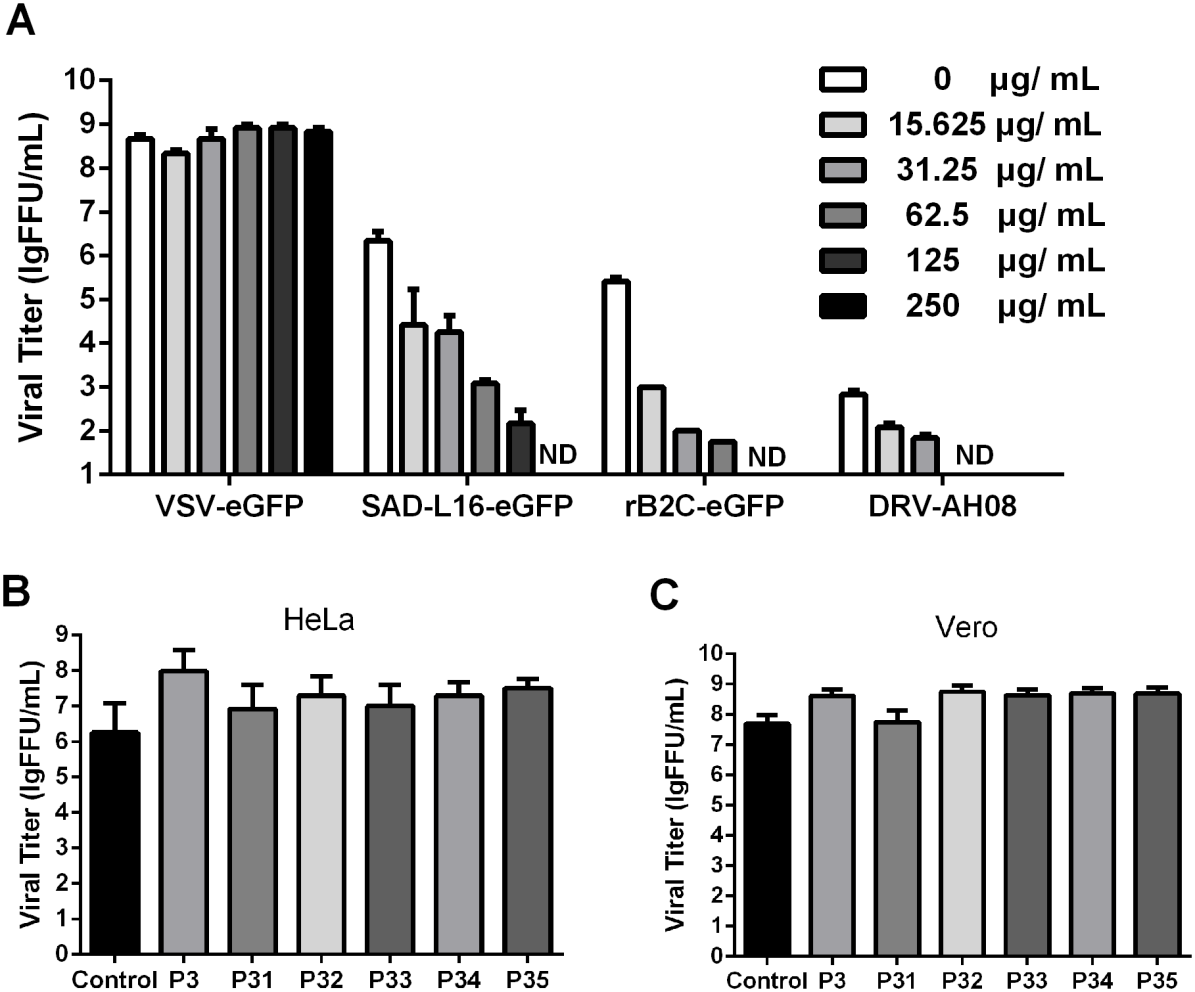
The inhibitory effects of P32 on VSV and different strains of RABV. (A) Confluent SK-N-SH cells were seeded in 24-well plates, and the viral titers of VSV and different strains of RABV were determined after incubating the cells with different concentrations of P32. Confluent HeLa cells (B) and Vero cells (C) were seeded in 24-well plates, and the viral titers of VSV were determined after incubating with different carrageenans (P3, P31, P32, P33, P34, and P35) at 250 μg/mL. Each value is expressed as the mean ± SEM from three independent experiments. ND means non-detectable.

To examine whether P32 could also inhibit different strains of RABV or VSV (another virus in the *Rhabdoviridae* family), three different RABV strains (SAD-L16-eGFP, rB2c-eGFP and DRV-AH08) and VSV-eGFP (vesicular stomatitis virus expressing GFP) were used for the viral inhibition assay. As shown in Fig 3, the viral replications of SAD-L16-eGFP, rB2c-eGFP and DRV-AH08 were fully inhibited with 250, 125 and 62.5 μg/mL P32, respectively, and viral inhibition occurred in a dose-dependent manner. In contrast, P32 (Fig 3A) and other carrageenans (P3, P31, P33, P34, and P35) did not affect the replication of VSV-eGFP in HeLa (Fig 3B), and Vero (Fig 3C) cell lines, indicating that the antiviral activity of P32 is specific for RABV and does not inhibit VSV infection.

### P32 inhibits RABV replication mainly during the viral post-adsorption stage

**Fig 4:**
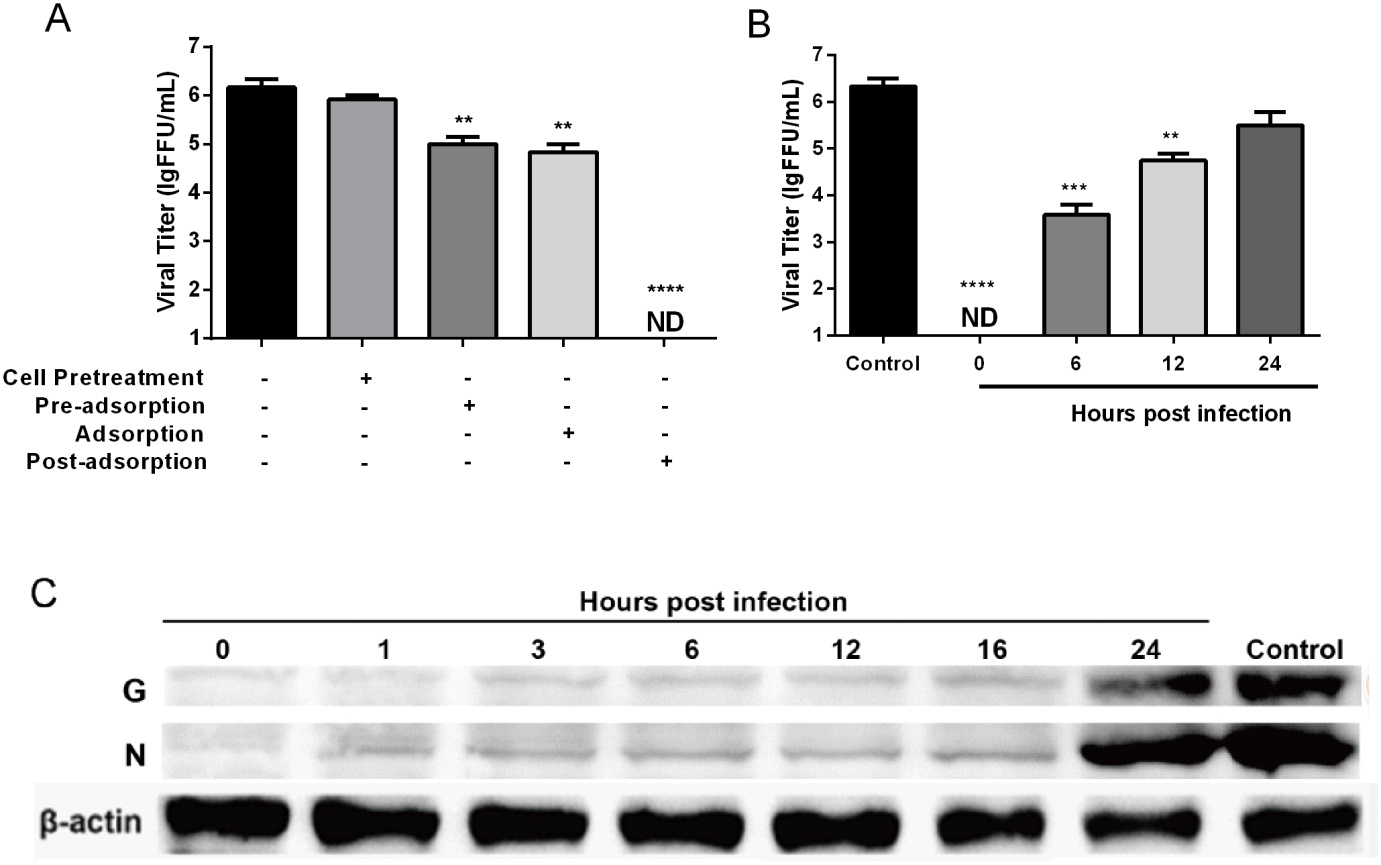
Inhibitory effects of P32 on RABV infection when administered under different conditions. (A) Inhibitory effects of P32 administered in the cell pre-treatment, pre-adsorption, adsorption, and post-adsorption stages. (B) Inhibitory effects of P32 administered at different time points during the viral post-adsorption period. SK-N-SH cells were infected with SAD-L16-eGFP (MOI = 0.01) and exposed to 250 μg/mL P32 in MM at the indicated time points. Culture supernatants were harvested at 48 hpi for virus titration. Each value is expressed as the mean ± SEM from three independent experiments (**, P<0.01; ***, P<0.001; ****, P<0.0005, ND means non-detectable). (C) Detection of nucleoprotein (N) and glycoprotein (G) levels of RABV by Western blot in RABV-infected cells that were treated with P32 at different time points during the viral post-adsorption period. NA cells were treated with 250 μg/mL P32 at the indicated time points after infection with the RABV strain SAD-L16 at an MOI of 0.01. At 48 hpi, cells were collected and lysed for Western blot analysis.

To determine whether P32 directly affects cell receptor, viral particles, viral adsorption and post-adsorption processes, viral inhibition assays were carried out on SK-N-SH cells by administering P32 under four different conditions, including cell pre-treatment (cells incubated with P32 before viral infection), pre-adsorption (virus was incubated with P32 before infecting cells), adsorption (virus was incubated with P32 after adsorbing to but not entering the cells) and post-adsorption (cells with internalized virus were incubated with P32). As shown in Fig 4A, compared with the control group (without P32 treatment), RABV replication was significantly inhibited by P32 in the pre-adsorption, adsorption, and post-adsorption groups. Notably, viral replication was completely inhibited in the post-adsorption group, indicating that P32 mainly affects RABV infection during the post-adsorption stage.

To further identify which step in the post-adsorption stage P32 affected most, the viral inhibition assay was performed at 0, 6, 12, and 24 h post-adsorption. As shown in Fig 4B, viral replication was significantly inhibited at 0, 6, and 12 h post-adsorption, and RABV replication was fully inhibited at 0 h post-adsorption. In addition, to further confirm the inhibition of viral replication, nucleoprotein (N) and glycoprotein (G) levels from RABV were detected at different time points post-adsorption by Western blotting. As shown in Fig 4C, the expression levels of N and G were obviously decreased when P32 was administered before 12 h post-adsorption or even 16 hpi, which is consistent with the results of the viral inhibition assay. Together, all these data indicate that P32 can inhibit RABV replication mainly by targeting the early stage (0–12 h) of the post-adsorption period.

### P32 affects RABV internalization

**Fig 5:**
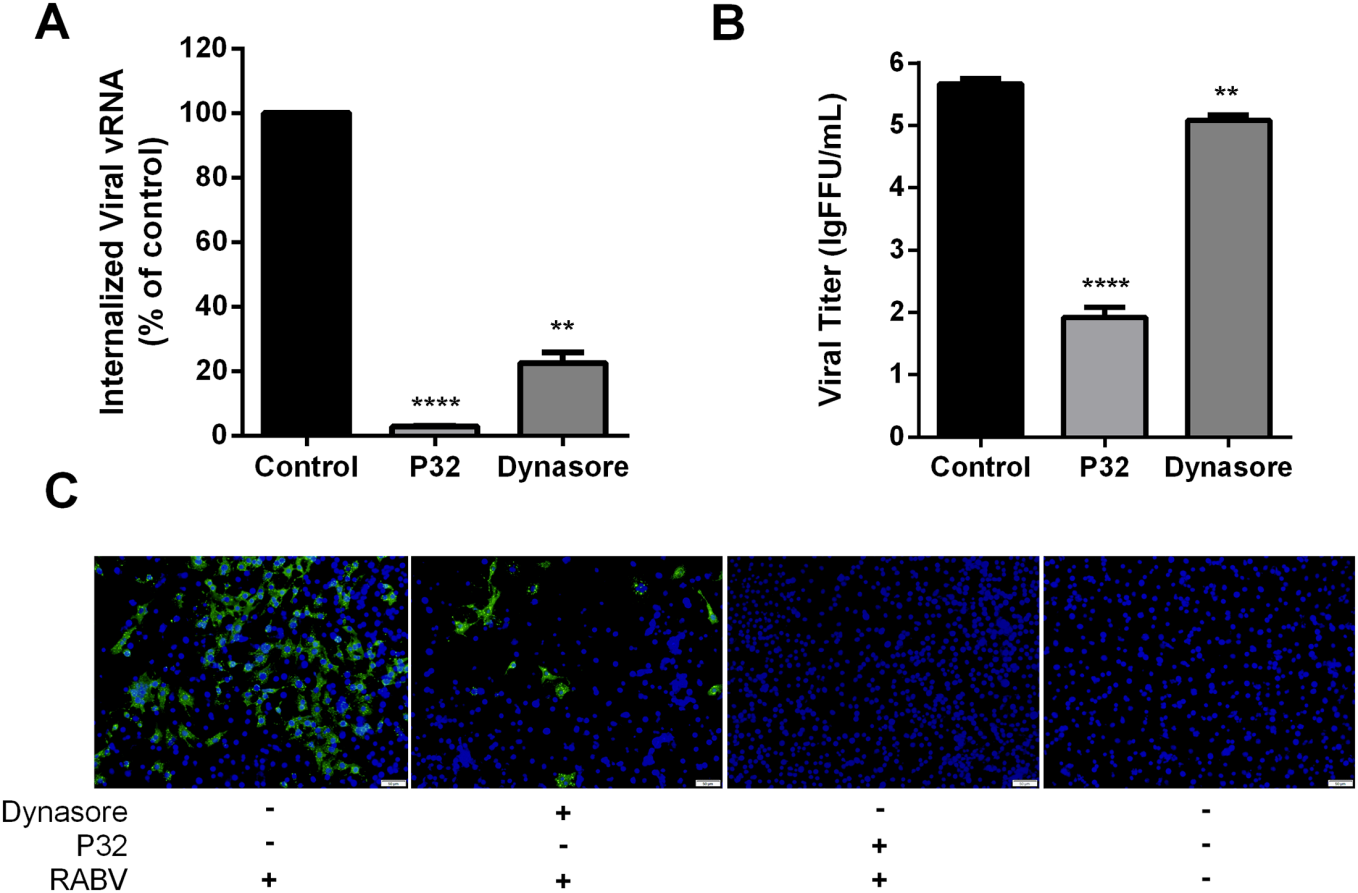
P32 inhibits RABV infection by interfering with viral internalization. (A) RNA levels of internalized RABV. SK-N-SH cells were infected with SAD-eGFP at an MOI of 5 and subjected to the viral endocytosis assay. Total RNA was extracted using TRIzol, and the amount of internalized viral RNA was determined by qRT-PCR. (B) Virus titer and (C) expression of RABV N protein after viral internalization with the addition of P32 or dynasore. For virus titer determination, BSR cells were infected with SAD-eGFP at an MOI of 0.01, and the viral endocytosis assay was performed as described in the Methods. Supernatants were harvested for virus titration. Each value is expressed as the mean ± SEM from three independent experiments (*, P<0.05; **, P<0.01; ***, P<0.001; ****, P<0.0005). For the detection of RABV N protein, BSR cells were subjected to the viral endocytosis assay, 48 hours after which, an immunofluorescence assay was conducted. A fluorescence microscope was used to observe the RABV N protein (apple green), and the nuclei were stained with DAPI (blue). The scale bar represents 50 μm.

After viral adsorption, RABV infection proceeds with internalization and uncoating [32]. RABV enters into cells by clathrin-mediated endocytosis [33]. To further determine whether P32 affects RABV internalization, the viral endocytosis assay was conducted. Dynasore, an inhibitor of dynamin, which is required for the scission step of clathrin-mediated endocytosis [34], was employed as the positive control treatment for the inhibition of RABV internalization. Viral RNA (vRNA) was harvested before and after internalization and analyzed by qRT-PCR. Moreover, the viral titer and expression level of RABV N protein were analyzed at 48 hpi. As shown in Fig 5A and 5B, significant differences in viral RNA levels and viral titers were detected between the P32-treated group and the control group. Moreover, the inhibitory effects of P32 treatment were even stronger than that observed in the dynasore-treated cells. Consistently, the expression of RABV N protein was almost completely suppressed by P32, as shown in Fig 5C. Together, these results indicate that P32 affects RABV internalization.

### P32 affects RABV G protein-mediated cell fusion

**Fig 6:**
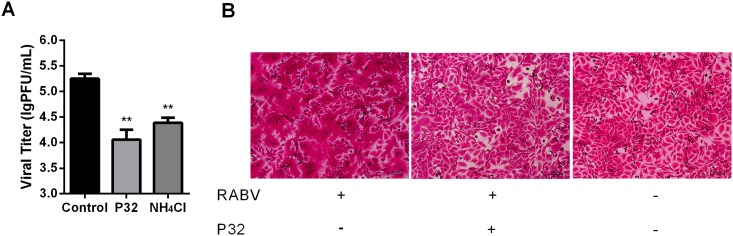
P32 affects cell fusion mediated by RABV G protein. (A) Viral titers in response to P32 treatment during the post-internalization period. NA cells were infected with SAD-L16 at an MOI of 0.01 and subjected to the post-internalization inhibition assay, after which viral titers were determined. Each value is expressed as the mean ± SEM from three independent experiments (*, P<0.05; **, P<0.01; ***, P<0.001; ****, P<0.0005). (B) P32 inhibits G protein-mediated cell membrane fusion. The low pH-dependent membrane fusion assay was performed in RABV-infected BSR cells. Briefly, BSR cells were infected with SAD-L16 at an MOI of 5 at 37°C for 48 h. After viral infection, cells were treated with MM containing P32 or vehicle at 37°C for 2 h, after which the cells were washed and incubated with fusion medium for 2 min at room temperature. Then, the fusion medium was discarded, and MM was added to the cells, which were incubated at 37°C for another 2 h. The cells were then fixed with 80% ice-cold acetone and stained with fuchsine solution.

RABV enters into cells via the endocytic pathway. A conformational change of RABV glycoproteins occurs in the acidic environment of the endosome (G), which results in the fusion of the viral envelope with the cellular membrane and culminates in viral uncoating [35]. Because λ-CG has been reported to block viral uncoating [15, 36], we next investigated whether P32 could affect RABV uncoating (cell fusion mediated by viral G protein). The post-internalization assay was performed to determine whether P32 could interfere the post-internalization events. Ammonium chloride, which can inhibit the acidification of endosomes, was employed as a positive control treatment for the inhibition of cell fusion [37, 38]. As shown in Fig 6A, the viral titer in the group treated with P32 or ammonium chloride was significantly lower than that of the control group, indicating that P32 could affect post-viral internalization events, including viral uncoating. To further confirm that P32 could affect viral uncoating by interfering with cell fusion mediated by RABV G protein, the low pH-dependent cell fusion assay was conducted as described in the Methods section. As shown in Fig 6B, cell fusion was obviously inhibited when cells were incubated with P32, whereas cell fusion was observed in most untreated RABV-infected cells, suggesting that P32 could affect cell fusion mediated by RABV G protein, which inhibits RABV uncoating.

### The inhibitory effect of P32 on RABV infection is stronger than heparan sulfate (HS) and heparin

**Fig 7:**
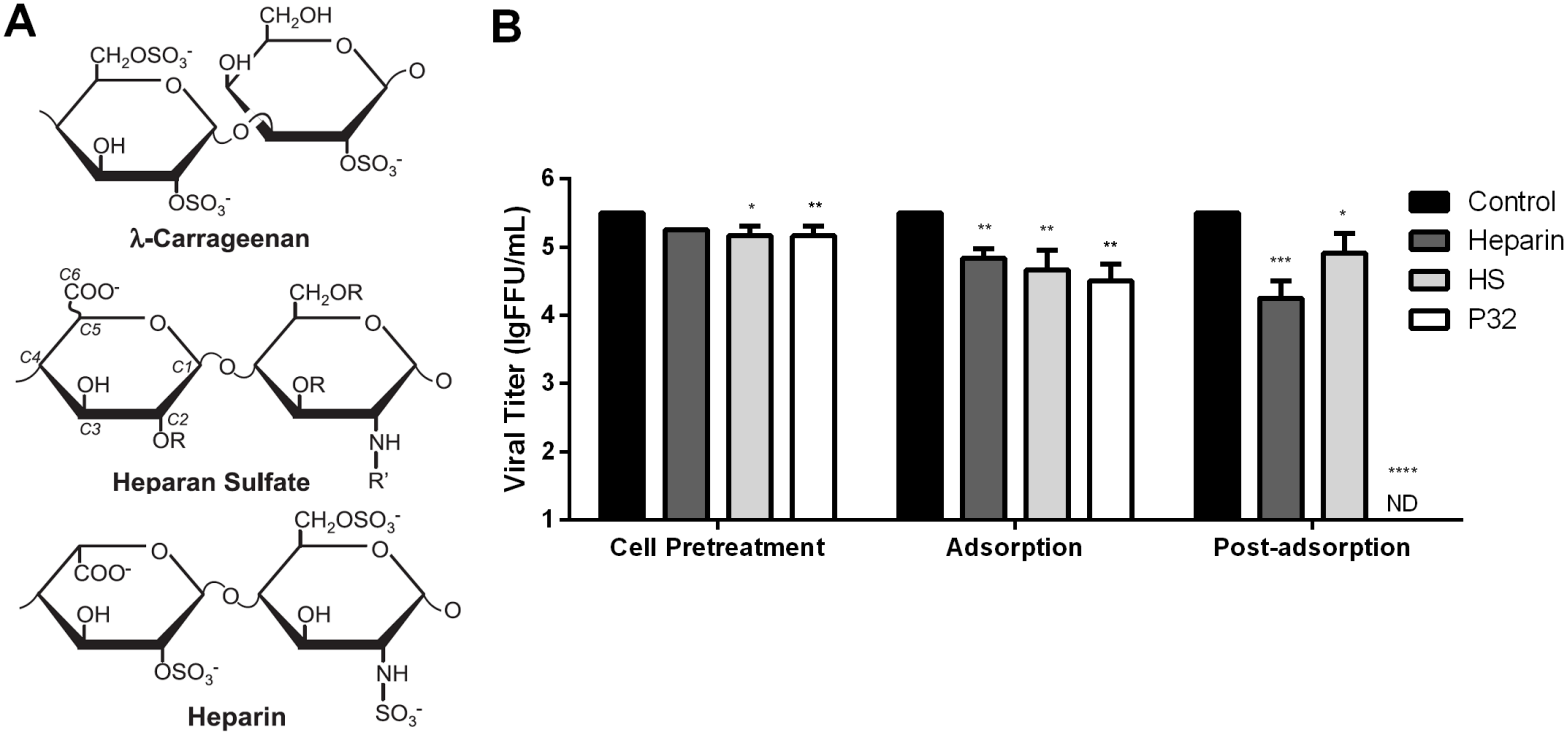
Comparison of the chemical structures and the anti-RABV infection effects among P32 and two sulfated polysaccharides. (A) Chemical structures of P32, HS and heparin [15]. (B) Inhibitory effects of P32, HS and heparin on RABV infection in the cell pre-treatment, adsorption and post-adsorption stages. SK-N-SH cells were infected with SAD-L16-eGFP, and the inhibition assay was carried out as described in the Methods section. Each value is expressed as the mean ± SEM from three independent experiments. ND means not detectable. (*, P<0.05; **, P<0.01; ***, P<0.001; ****, P<0.0005).

Previous studies have demonstrated that carrageenan blocks viral infection based on its structure, which is similar to a cell surface glycosaminoglycan HS [36, 39]. Moreover, the efficiency of viral inhibition is closely connected to sulfate chemical groups [40]. To investigate whether other compounds with chemical structures similar to HS could also inhibit RABV infection like P32, viral inhibition assays were performed on SK-N-SH cells using HS, heparin (a highly sulfated form of HS) and P32. The chemical structures of these three compounds are very similar, as listed in Fig 7A. We observed significant inhibitory effects for all three compounds when administered to cells during the pre-treatment, adsorption and post-adsorption stages, with the exception of heparin treatment during the pre-treatment stage (Fig 7B). Of note, P32 had the strongest inhibitory effect on RABV infection of the three compounds for all different assays. Moreover, P32 completely inhibited viral infection when administered during the post-adsorption assay, which is consistent with our previous result.

Together, our results indicate that λ-CG P32 is a promising inhibitor of RABV infection that affects viral uncoating during the viral post-adsorption stage.

## Discussion

Rabies remains a major threat to public health that causes more than 55,000 human deaths each year worldwide. Currently, no effective therapy is available for rabies once clinical signs appear. Developing anti-RABV drugs is critical for the elimination of rabies.

In this study, the carrageenan P32 was investigated for its ability to inhibit RABV infection. Carrageenan is a selective inhibitor of several viruses, including both enveloped viruses [19, 36, 41, 42] and naked viruses [15, 43]. Carrageenan can inhibit virus attachment to host cells mainly by binding with viral glycoproteins or envelope proteins. The function of carrageenan is possibly attributed to the fact that its chemical structure is similar to HS, a cell surface receptor of many viruses, such as dengue virus, herpesvirus, human enterovirus 71, human immunodeficiency virus (HIV) and human papillomavirus [15, 36, 41, 44–46]. Carrageenan can also block infection by attaching to cell membrane receptors targeted by viruses or by inhibiting viral transcription and replication [42, 47–49]. Our results indicate that a λ-CG P32 can inhibit RABV infection by affecting viral internalization and cell fusion mediated by RABV G protein, which blocks viral uncoating. A previous study demonstrated that carrageenan could inhibit viral uncoating during dengue virus infection [36].

RABV is a member of the *Rhabdoviridae* family. To investigate whether P32 can also inhibit other viruses within the family, the inhibitory effect of P32 for vesicular stomatitis virus (VSV) infection was tested. Our results show that P32 could inhibit both fixed and wild-type RABV infection rates; however, almost no inhibitory effect was observed for VSV infections. A previous study has shown that λ-CG could inhibit VSV infections [50], which is not consistent with our results. One possible reason for this discrepancy is that the λ-CG used in the previous study differs structurally from P32.

P32 inhibited RABV infection under four different conditions in our study. The strongest inhibitory effect was observed during the post-adsorption period, especially the early stages. A previous study demonstrated that carrageenan could affect viral internalization during H1N1 influenza virus infection [51]. Consistently, RABV internalization was also affected by P32 in our study. Furthermore, the low pH-dependent cell fusion assay performed in our study revealed that cell fusion mediated by RABV G protein was dramatically inhibited by P32, suggesting that P32 affects viral uncoating. A previous study demonstrated that carrageenan oligosaccharide could enter cells and affect viral replication [49], suggesting that P32 could inhibit the conformational changes of RABV G protein to block cell fusion mediated by G protein thereby inhibiting viral uncoating. Similar inhibitory action of carrageenan was observed previously in dengue virus [36].

Previous studies demonstrated that one possible mechanism for the viral infection inhibitory action of carrageenan is that the structure of carrageenan is similar to the cell surface glycosaminoglycan HS, which is a well-known receptor for many viruses, such as herpes simplex virus and pseudorabies virus [36, 39]. We conducted a comparison between P32 and the structural analogs of P32 (HS and heparin) and found that the inhibition effect of P32 on RABV infection was stronger than HS and heparin, suggesting that the remarkable anti-RABV activity of P32 may be attributable to more than its structural similarity to HS.

Carrageenan has been shown to be effective in reducing herpes simplex virus within the genital tract [16, 18]. Based on the novel inhibitory effects of λ-CG P32 on RABV infection, P32 could be developed as a candidate anti-RABV drug that could be used for wound cleaning, although safety issues remain to be investigated. In addition, the structure of P32 may provide new insights into the development of novel anti-RABV drugs.

Altogether, our results suggest that λ-CG P32 is a novel anti-RABV agent that can effectively inhibit RABV infection *in vitro* by affecting viral internalization and cell fusion mediated by viral G protein. P32 could be a promising candidate as an anti-RABV drug.
